# Supplementary material for: Osteoclast-associated receptor blockade prevents articular cartilage destruction via chondrocyte apoptosis regulation
Source: Nat Commun. 2020 Aug 28;11:4343. doi: 10.1038/s41467-020-18208-y (PMC7455568; doi:10.1038/s41467-020-18208-y)
Supplement: Supplementary file 1 — Supplementary Information [file 41467_2020_18208_MOESM1_ESM.pdf]

Supplementary Information

Osteoclast-associated receptor blockade prevents articular cartilage destruction via chondrocyte apoptosis regulation

Park et al.

This file contains:

Supplementary Figures 1-5

Supplementary Tables 1-2

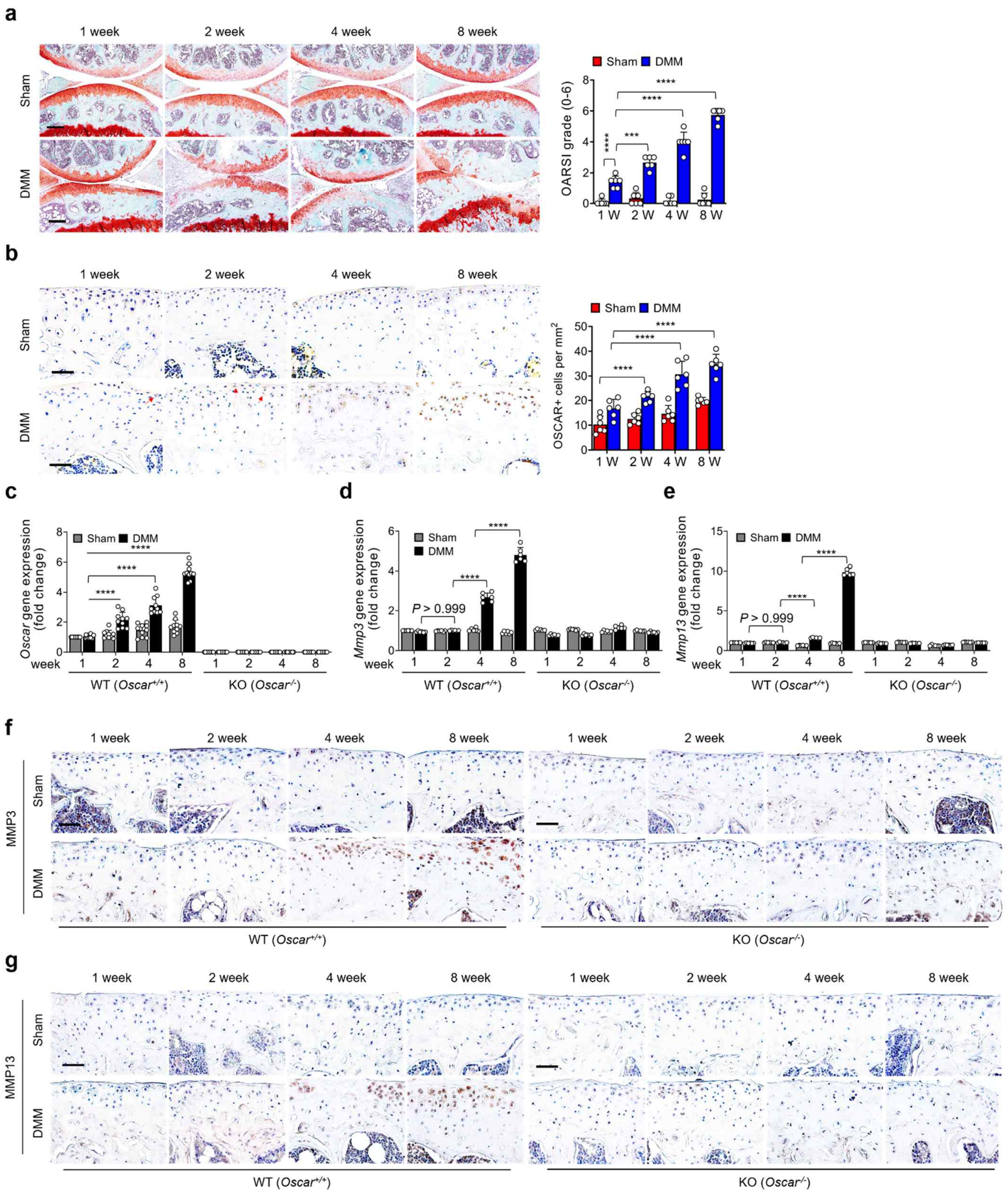

Supplementary Figure 1. Expression of OSCAR during OA development. (a) Experimental OA was examined by safranin-O staining and scoring of OA parameters, including articular cartilage destruction (OARSI grade) in WT and *Oscar*$^{-/-}$ mice. Bar graphs display mean ± S.E.M. for *n* = 6 mice. *Oscar*$^{-/-}$and WT mice underwent sham or DMM surgery. Scale bar = 200 μm. **(b)** IHC analyses of OSCAR in OA articular cartilage from DMM surgery mice

compared to sham-operated mice (mean ± S.E.M. for $n = 6$). Red arrowheads indicate OSCAR-positive cells. Scale bar = 50 μm. **(c-e)** qRT-PCR analysis of articular cartilage tissues of WT and *Oscar*$^{-/-}$ mice subjected to sham or DMM surgery. Error bars represent mean ± S.E.M. of $n = 10$ mice for *Oscar*, $n = 6$ for *Mmp*3, and *Mmp13*. **(f, g)** IHC of MMP3 **(f)** and MMP13 **(g)** in OA articular cartilage from DMM surgery mice compared to sham-operated mice ($n = 6$). Scale bar = 50 μm. Two-way ANOVA was performed followed by Sidak's Multiple Comparison's test, with p-values indicated in figure. ***$P = 0.001$, ****$P < 0.0001$.

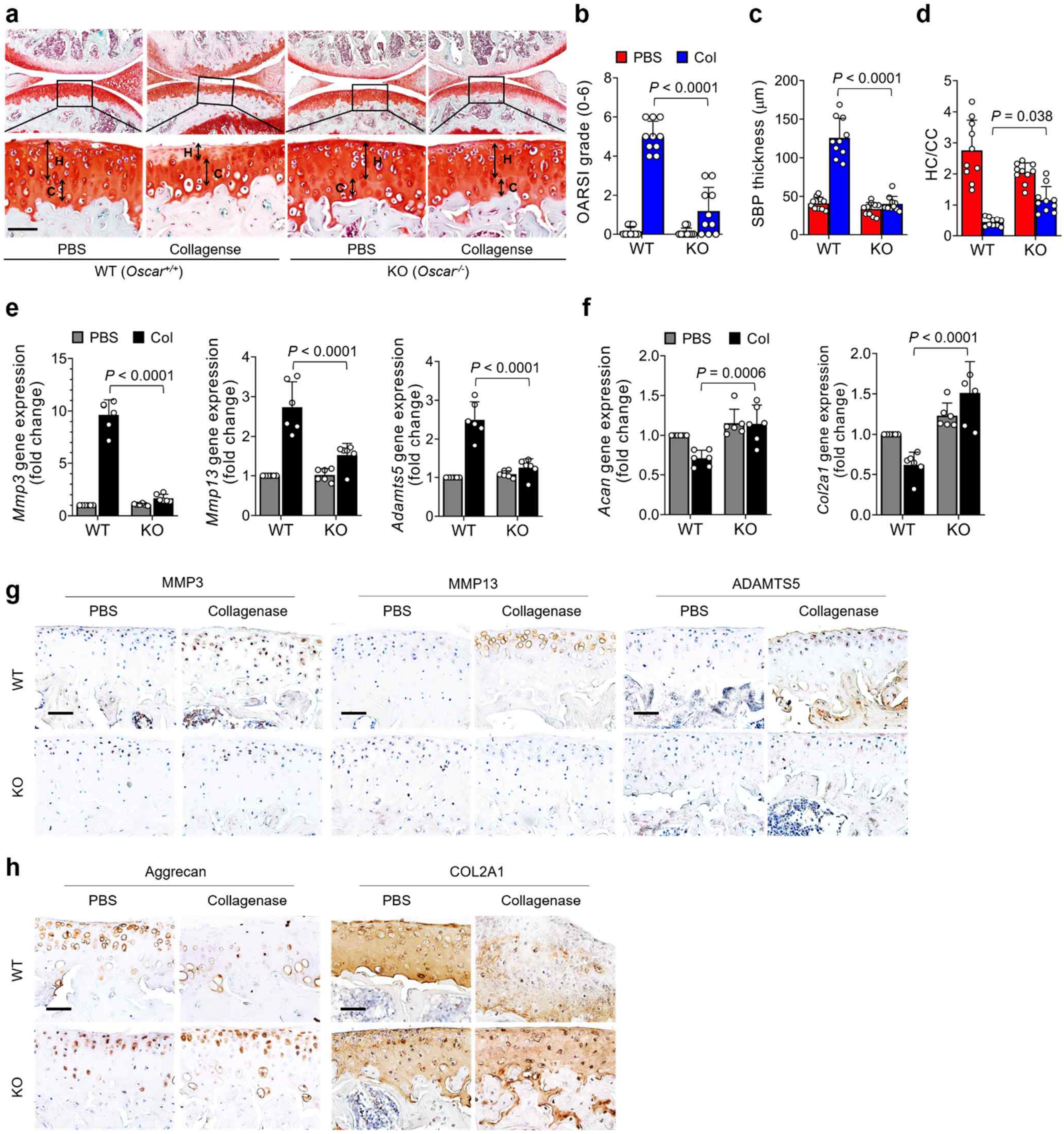

Supplementary Figure 2. Deletion of OSCAR protects articular cartilage destruction in collagenase-induced OA. (a–d) Experimental OA was examined by safranin-O staining (**a**) and scoring of OA parameters, including articular cartilage destruction (OARSI grade) (**b**), SBP thickness **(c)**, and ratio of hyaline cartilage (HC) and calcified cartilage (CC) **(d)** in WT and *Oscar*$^{-/-}$ mice. *Oscar*$^{-/-}$ and WT mice injected with collagenase (Col) or PBS. Scale bar = 50 μm. Error bars represent mean ± S.E.M. of n = 10 mice (**b-d**). **(e, f)** qRT-PCR analysis in articular cartilage tissues of WT and *Oscar*$^{-/-}$ mice injected with collagenase or PBS with error bars representing mean ± S.E.M. of n = 6**. (g, h)** IHC analyses of MMP3, MMP13, and ADAMTS5 **(g),** aggrecan and COL2A1 (h) in OA articular cartilage from WT and *Oscar*$^{-/-}$ mice injected with collagenase or PBS (n = 10). Scale bar = 50 μm. Two-way ANOVA was performed followed by Sidak's Multiple Comparison's test, with p-values indicated in figure.

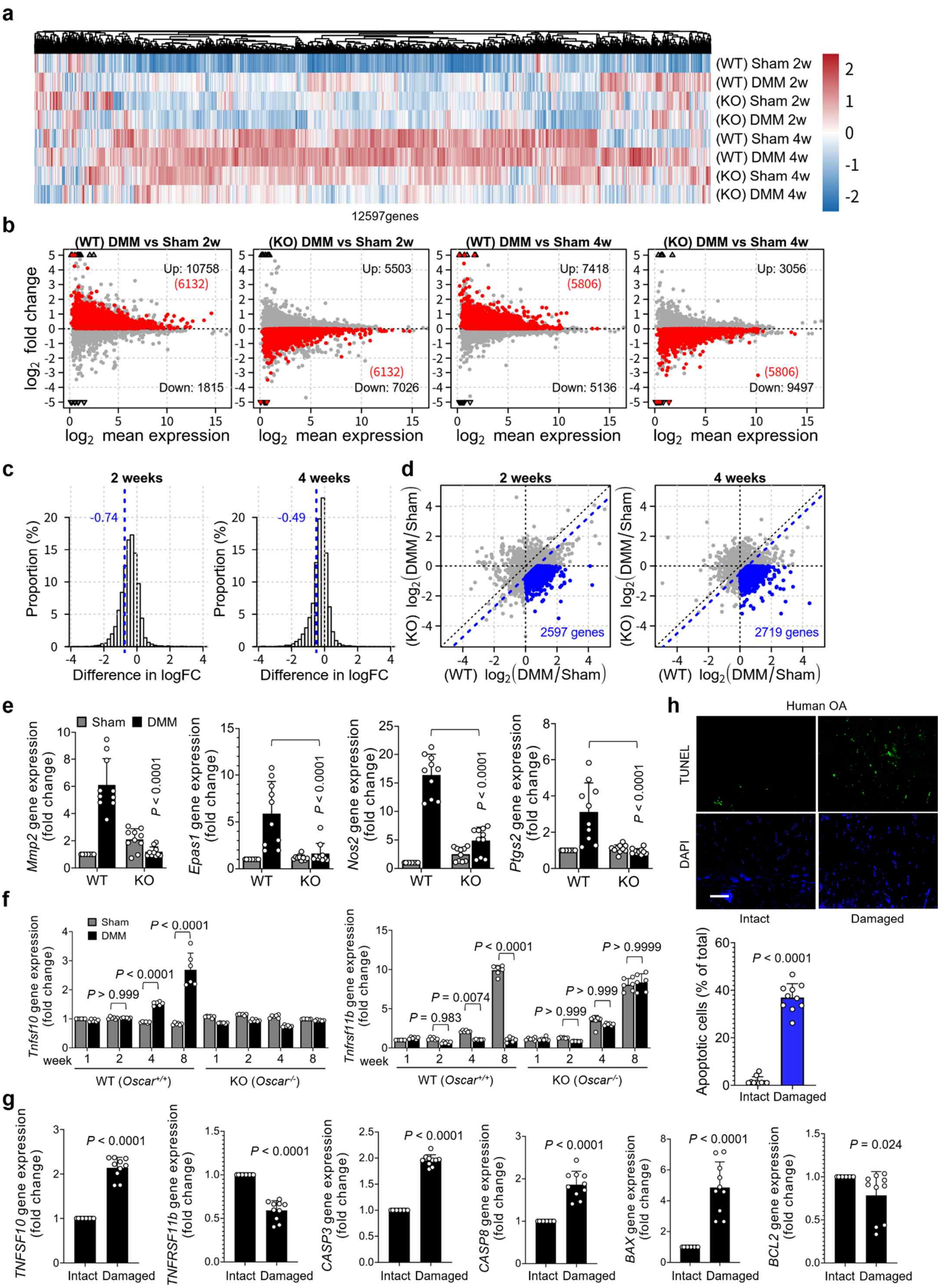
a
(WT) Sham 2w
(WT) DMM 2w
(KO) Sham 2w
(KO) DMM 2w
(WT) Sham 4w
(WT) DMM 4w
(KO) Sham 4w
(KO) DMM 4w
12597genes
b
(WT) DMM vs Sham 2w
(KO) DMM vs Sham 2w
(WT) DMM vs Sham 4w
(KO) DMM vs Sham 4w
Up: 10758
(6132)
Down: 1815
Up: 5503
(6132)
Down: 7026
Up: 7418
(5806)
Down: 5136
Up: 3056
(5806)
Down: 9497
log2 fold change
log2 mean expression
c
2 weeks
4 weeks
-0.74
-0.49
Proportion (%)
Difference in logFC
d
2 weeks
4 weeks
(KO) log2(DMM/Sham)
(WT) log2(DMM/Sham)
2597 genes
2719 genes
e
Sham
DMM
Mmp2 gene expression (fold change)
Epas1 gene expression (fold change)
Nos2 gene expression (fold change)
Ptgs2 gene expression (fold change)
P < 0.0001
WT
KO
h
Human OA
TUNEL
DAPI
Intact
Damaged
Apoptotic cells (% of total)
P < 0.0001
f
Tnfsf10 gene expression (fold change)
Tnfrsf11b gene expression (fold change)
P > 0.999
P < 0.0001
P = 0.983
P = 0.0074
P > 0.9999
week
WT (Oscar+/+)
KO (Oscar-/-)
g
TNFSF10 gene expression (fold change)
TNFRSF11b gene expression (fold change)
CASP3 gene expression (fold change)
CASP8 gene expression (fold change)
BAX gene expression (fold change)
BCL2 gene expression (fold change)
P = 0.024
Intact Damaged

Supplementary Figure 3. OSCAR regulates apoptotic signaling in OA-induced articular cartilage. (a) Gene-wise scaled (z-score transformed) gene expression (TPM) values for all genes in each sample (*n* = 10). **(b)** MA plot showing the gene expression changes between DMM- and sham-operated mice. Each plot (from left to right) shows expression changes in WT and *Oscar*$^{-/-}$ mice at 2 and 4 weeks after surgery, respectively. Genes upregulated in WT mice but down-regulated in *Oscar*$^{-/-}$ mice at each time point (2 or 4 weeks) are labeled in red. **(c)** Distribution of 12,597 DIF scores derived from RNA seq data at 2 and 4 weeks, respectively. The lower quartile value (DIF25) is indicated by a blue line. **(d)** Scatter plot of log2-transformed fold-change between DMM- and sham-operated mice (log_2FC) in WT mice versus *Oscar*$^{-/-}$ mice. Genes with a lower DIF score than DIF25 are colored in blue. **(e)** qRT-PCR analyses of hypoxia and other genes in articular cartilage tissues of WT and *Oscar*$^{-/-}$ mice subjected to sham or DMM surgery. Error bars represent mean ± S.E.M. of *n* = 10 mice. **(f)** qRT-PCR analyses in articular cartilage tissues of WT and *Oscar*$^{-/-}$mice subjected to sham or DMM surgery at time intervals as indicated. Error bars represent mean ± S.E.M. of *n* = 6 mice. **(g)** qRT-PCR analyses of intact and damaged regions of human OA articular cartilage. Error bars represent mean ± S.E.M. of *n* = 10 patient samples. **(h)** Apoptotic articular chondrocytes were detected and quantified by TUNEL assay representing mean ± S.E.M. of *n* = 10 patient samples. Scale bar = 200 μm. Two-way ANOVA was performed followed by Sidak's Multiple Comparison's test **(e, f)** and two-tailed student's *t*-test was performed **(g, h)**, with *p*-values indicated in figure.

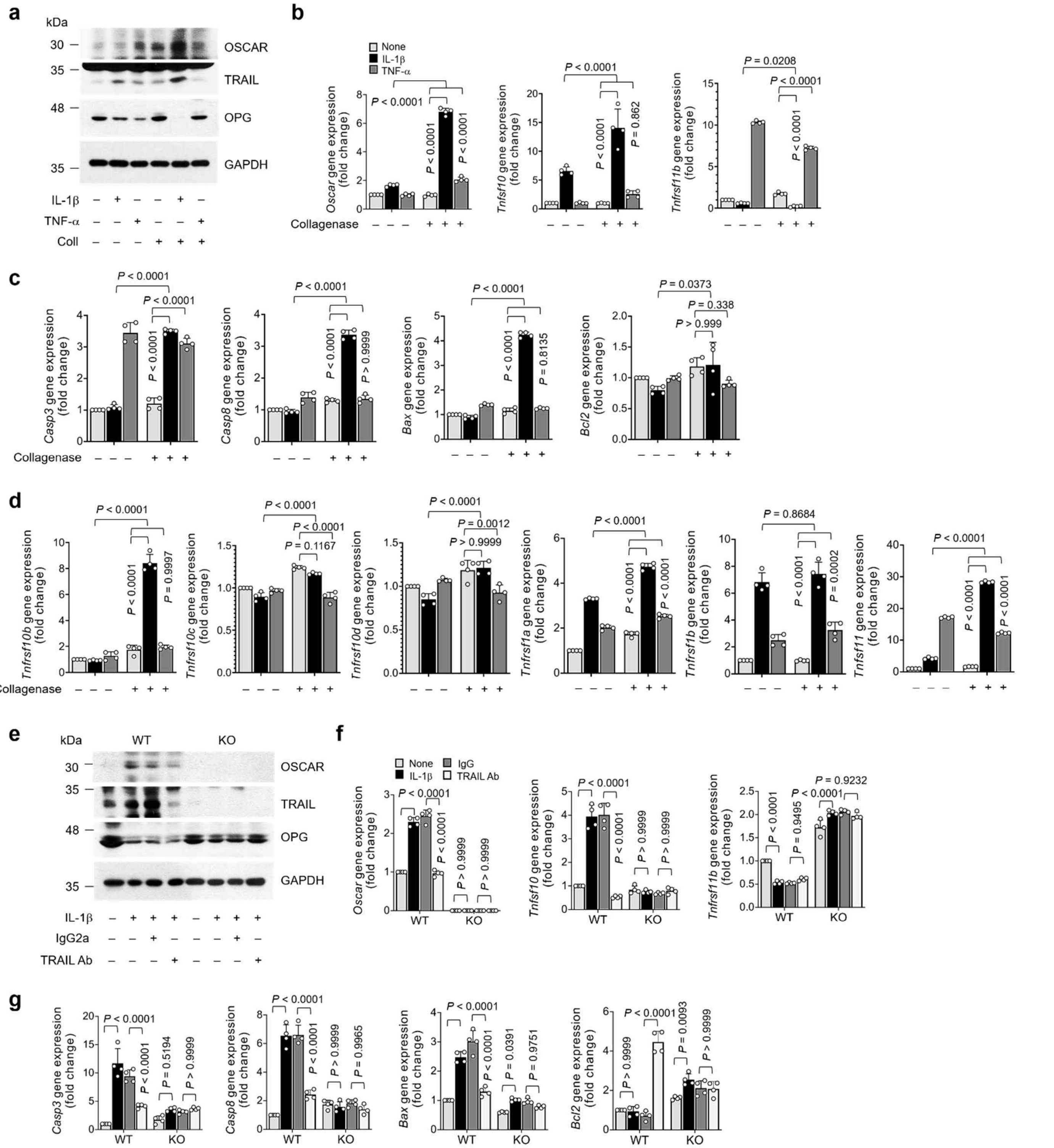

Supplementary Figure 4. Effects of pro-inflammatory cytokines with collagenase on the expression of apoptosis regulatory genes. (a-e) Western blot analysis **(a)** and qRT-PCR analysis **(b-d)** in mouse articular chondrocytes treated with IL-1β (5 ng mL^{-1}) or TNF-α (50 ng mL^{-1}) and collagenase (50 U mL^{-1}) for 48 h. Error bars represent mean ± S.E.M. for n = 4 independent experiments. **(e-g)** Western blotting **(e)** and qRT-PCR analysis **(f,g)** in articular chondrocytes of WT and *Oscar*$^{-/-}$ mice treated with TRAIL neutralizing antibody (1 μg mL^{-1}) before IL-1β treatment. Bar graphs shown in **(f, g)** are mean ± S.E.M. for n = 4 independent experiments. Two-way ANOVA was performed followed by Sidak's Multiple Comparison's test, with p-values indicated in figure.

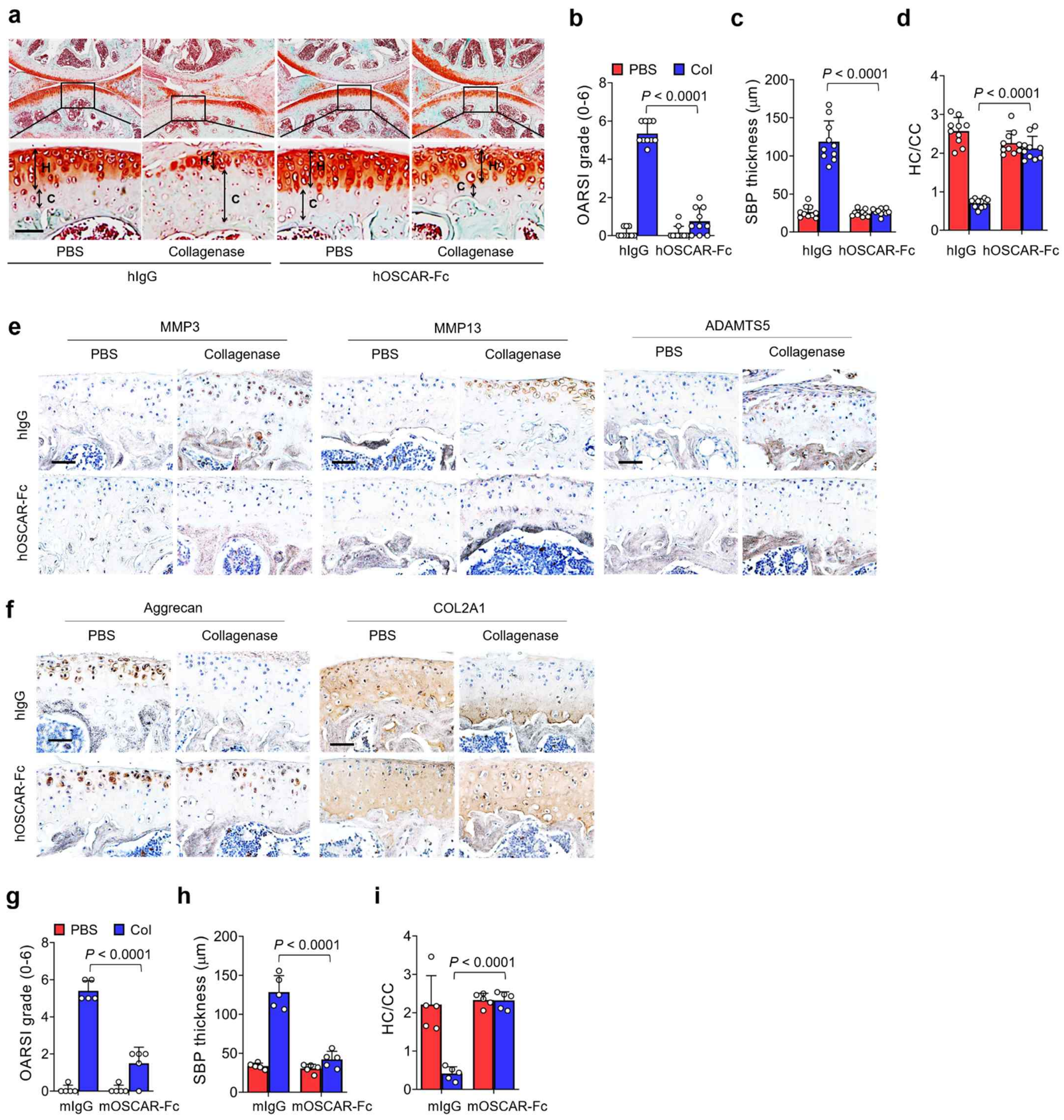

Supplementary Figure 5. OSCAR-Fc blocks articular cartilage degeneration in mice with induced OA. (a–d) WT mice injected with PBS or collagenase (Col) were IA-injected with human IgG as a control or hOSCAR-Fc (2 mg kg^{-1}) to block OSCAR in joint tissues. Articular cartilage sections were subjected to safranin-O staining **(a)** and quantitative analysis of OARSI grade **(b)**, SBP thickness **(c)**, and ratio of hyaline cartilage (HC) and calcified cartilage (CC) calculated **(d)**. Scale bar = 50 μm**.** Error bars represent mean ± S.E.M. of n = 10 mice (**b-d**). **(e, f)** IHC analyses of MMP3, MMP13, and ADAMTS5 **(e),** aggrecan and COL2A1 **(f)** in OA articular cartilage from WT mice injected with collagenase or PBS (n = 10). Scale bar = 50 μm. **(g–i)** WT mice injected with collagenase (Col) or PBS were IA-injected with mouse IgG as a control or mOSCAR-Fc (2 mg kg^{-1}) to block OSCAR in joint tissues. Articular cartilage sections were subjected to quantitative analysis of OARSI grade **(g)**, SBP (SBP Th) thickness **(h)**, and ratio of hyaline cartilage (HC) and calcified cartilage (CC) **(i)**. Error bars represent mean ± S.E.M. of n = 5 mice (**g-i**). Two-way ANOVA was performed followed by Sidak's Multiple Comparison's test, with p-values indicated in figure.

Supplementary Table1. *Oscar* siRNA sequences.

Gene	Strand	Primer sequence
si*Oscar a*	Sense<br>Antisense	5’-CCUACUGUUGCUAUUACCAUU-3’<br>5’-UGGUAAUAGCAACAGUAGGUU-3’
si*Oscar b*	Sense<br>Antisense	5’-CUAAUGUUAGUGACUUUAUUU-3’<br>5’-AUAAAGUCACUAACAUUAGUU-3’
si*Oscar c*	Sense<br>Antisense	5’-CUUUAUCCUCCCUUGAAAAUU-3’<br>5’-UUUUCAAGGGAGGAUAAAGUU-3’
Control siRNA	Sense<br>Antisense	5’-CCUCGUGCCGUUCCAUCAGGUAGUU-3’<br>5’-CUACCUGAUGGAACGGCACGAGGUU-3’

Supplementary Table2. Oligonucleotides used for real time PCR analysis.

Gene	Strand	Primer sequence	Origin
Oscar	Sense<br>Antisense	5’-AGGGAAACCTCATCCGTTT-3’<br>5’-TGCTGTGCCAATCACAAGTA-3’	Mouse
OSCAR	Sense<br>Antisense	5’-GCTTCATACCACCCTAAGCC-3’<br>5’-AAAGTCCAAATCTCCAAGCG-3’	Human
Mmp3	Sense<br>Antisense	5’-TCCTGATGTTGGTGGCTTCAG-3’<br>5’-TGTCTTGGCAAATCCGGTGTA-3’	Mouse
Mmp13	Sense<br>Antisense	5’-CCTTGAACGTCATCATCAGG-3’<br>5’-TGTTTATTGTTGCTGCCCAT-3’	Mouse
Adamts5	Sense<br>Antisense	5’-GCCATTGTAATAACCCTGCACC-3’<br>5’-TCAGTCCCATCCGTAACCTTTG-3’	Mouse
Acan	Sense<br>Antisense	5’-GAAGACGACATCACCATCCAG-3’<br>5’-CTGTCTTTGTCACCCACACATG-3’	Mouse
Col2a1	Sense<br>Antisense	5’-CACACTGGTAAGTGGGGCAAGA-3’<br>5’-GGATTGTGTTGTTTCAGGGTTCG-3’	Mouse
Tnfsf10	Sense<br>Antisense	5’-CCTCTCGGAAAGGGCATTC-3’<br>5’-TCCTGCTCGATGACCAGCT-3’	Mouse
TNFSF10	Sense<br>Antisense	5’-GAGTATGAACAGCCCCT-3’<br>5’-GTTGCTTCTTCCTCTGGT-3’	Human
Tnfrsf11b	Sense<br>Antisense	5’-CAGAGCGAAACACAGTTTG-3’<br>5’-CACACAGGGTGACATCTATTC-3’	Mouse
TNFRSF11B	Sense<br>Antisense	5’-TCCTGGATTTGGAGTGGTGC-3’<br>5’-AGCAGGAGACCAAAGACACT-3’	Human
Mmp2	Sense<br>Antisense	5’-CCAACTACGATGATGAC-3’<br>5’-ACCAGTGTCAGTATCAG-3’	Mouse
Epas1	Sense<br>Antisense	5’-CGAGAAGAACGACGTGGTGTTC-3’<br>5’-GTGAAGGCGGGCAGGCTCC-3’	Mouse
Nos2	Sense<br>Antisense	5’-ACCTTGTTCAGCTACGCCTT-3’<br>5’-CATTCCCAAATGTGCTTGTC-3’	Mouse
Ptgs2	Sense<br>Antisense	5’-CTCAATGAGTACCGCAAACG-3’<br>5’-TTCAATTCTGCAGCCATTTC-3’	Mouse
Casp3	Sense<br>Antisense	5’-GATCTTACTCGTGAAGACATTTTGGAA-3’<br>5’-CTTCATCACCATGGCTTAGAATCAC-3’	Mouse
CASP3	Sense<br>Antisense	5’-GAGCTGCCTGTAACTTG-3’<br>5’-ACCTTTAGAACATTTCCACT-3’	Human
Casp8	Sense<br>Antisense	5’-CTGGTCAACTTCCTAGACTGCAA-3’<br>5’-ATCTCAATTCCAACTCGCTCACT-3’	Mouse
CASP8	Sense<br>Antisense	5’-AGAGTCTGTGCCCAAATCAAC-3’<br>5’-GCTGCTTCTCTCTTTGCTGAA-3’	Human
Bax	Sense<br>Antisense	5’-GAACAGATCATGAAGACAGGG-3’<br>5’-CAGTTCATCTCCAATTCGCC-3’	Mouse

BAX	Sense<br>Antisense	5’-CCCGAGAGGTCTTTTTCCGAG-3’<br>5’-CCAGCCCATGATGGTTCTGAT-3’	Human
Bcl2	Sense<br>Antisense	5’-TGTGGATGACTGAGTACCTGAAC-3’<br>5’-GAGAAATCAAACAGAGGTCGCATG-3’	Mouse
BCL2	Sense<br>Antisense	5’-ACGAGTGGGATGGGGGAGATGTG-3’<br>5’-GCGGTAGCGGCGGGAGAAGTC-3’	Human
Tnfrsf10b	Sense<br>Antisense	5’-TTGGAATGGCTGGTGTAGTC-3’<br>5’-TGCTGCTTGCTGTGCTAC-3’	Mouse
Tnfrsf10c	Sense<br>Antisense	5’-TTTCCGGAATCATGCCGCCCA-3’<br>5’-AGGACCAGCCAGTTTCTGGGATTTG-3’	Mouse
Tnfrsf10d	Sense<br>Antisense	5’-TGGCTTCTTCTGCAGCTTGGTGT-3’<br>5’-GGGATTTCGCAGGGCGCCTT-3’	Mouse
Tnfrsf1a	Sense<br>Antisense	5’-GGGCACCTTTACGGCTTCC-3’<br>5’-GGTTCTCCTTACAGCCACACA-3’	Mouse
Tnfrsf1b	Sense<br>Antisense	5’-GTCGCGCTGGTCTTCGAACTG-3’<br>5’-GGTATACATGCTTGCCTCACAGTC-3’	Mouse
Tnfsf11	Sense<br>Antisense	5’-ATCGGGAAGCGTACCTACAG-3’<br>5’-GTGCTCCCTCCTTTCATCAG-3’	Mouse
β-Actin	Sense<br>Antisense	5’-TGGAATCCTGTGGCATCCATGAAAC -3’<br>5’-TAAAACGCAGCTCAGTAACAGTCCG-3’	Mouse
β-ACTIN	Sense<br>Antisense	5’-GCCGGGACCTGACTGACTAC -3’<br>5’-TTCTCCTTAATGTCACGCACGAT-3’	Human
