# Supplementary material for: Osteoclast-associated receptor blockade prevents articular cartilage destruction via chondrocyte apoptosis regulation
Source: Nat Commun. 2020 Aug 28;11:4343. doi: 10.1038/s41467-020-18208-y (PMC7455568; doi:10.1038/s41467-020-18208-y)
Supplement: Supplementary file 3 — Reporting Summary [file 41467_2020_18208_MOESM3_ESM.pdf]

nature research

Corresponding author(s): Soo Young Lee

Last updated by author(s): May 4, 2020

Reporting Summary

Nature Research wishes to improve the reproducibility of the work that we publish. This form provides structure for consistency and transparency in reporting. For further information on Nature Research policies, seeAuthors & Referees and theEditorial Policy Checklist .

Statistics

For all statistical analyses, confirm that the following items are present in the figure legend, table legend, main text, or Methods section.

n/a	Confirmed	
☐	☒	The exact sample size (*n*) for each experimental group/condition, given as a discrete number and unit of measurement
☐	☒	A statement on whether measurements were taken from distinct samples or whether the same sample was measured repeatedly
☐	☒	The statistical test(s) used AND whether they are one- or two-sided<br>*Only common tests should be described solely by name; describe more complex techniques in the Methods section.*
☐	☒	A description of all covariates tested
☐	☒	A description of any assumptions or corrections, such as tests of normality and adjustment for multiple comparisons
☐	☒	A full description of the statistical parameters including central tendency (e.g. means) or other basic estimates (e.g. regression coefficient) AND variation (e.g. standard deviation) or associated estimates of uncertainty (e.g. confidence intervals)
☐	☒	For null hypothesis testing, the test statistic (e.g. *F*, *t*, *r*) with confidence intervals, effect sizes, degrees of freedom and *P* value noted<br>*Give P values as exact values whenever suitable.*
☒	☐	For Bayesian analysis, information on the choice of priors and Markov chain Monte Carlo settings
☒	☐	For hierarchical and complex designs, identification of the appropriate level for tests and full reporting of outcomes
☒	☐	Estimates of effect sizes (e.g. Cohen's *d*, Pearson's *r*), indicating how they were calculated

Our web collection on statistics for biologists contains articles on many of the points above.

Software and code

Policy information about availability of computer code

Data collection	Image analysis: Olympus DP72, OsteoMeasure XP.version 2.0.0.2, ~~Media Cybernetics Image pro plus 4.5~~<br>qRT-PCR analysis: StepOne™ Software, version. 2.3
Data analysis	Olympus DP72<br>OsteoMeasure XP, version 2.0.0.2<br>~~Media Cybernetics Image pro plus 4.5~~<br>StepOne™ Software, version. 2.3<br>GraphPad Prism, version 8.1.2 (332)<br>Adobe photoshop 19.1.3<br>SoftMax pro 5.4.1<br>~~RAN~~ sequencing analysis data was using STAR aligner (version2.6.0a), and normalized TPM (Transcripts Per Kilobase Million) values of each gene were calculated using RSEM (version 1.2.17) with Gencode v22 annotation.<br>Micro CT analysis data was using Skyscan 1176, and CT Analyzer.

For manuscripts utilizing custom algorithms or software that are central to the research but not yet described in published literature, software must be made available to editors/reviewers. We strongly encourage code deposition in a community repository (e.g. GitHub). See the Nature Research guidelines for submitting code & software for further information.

Data

Policy information about availability of data

All manuscripts must include a data availability statement. This statement should provide the following information, where applicable:

- Accession codes, unique identifiers, or web links for publicly available datasets
- A list of figures that have associated raw data
- A description of any restrictions on data availability

The data that support the findings of this study are available within the article and its Supplementary Information files or from the corresponding author upon reasonable request. RNA seq of articular cartilage in WT and Oscar-/- mice can be obtained from Gene Expression Omnibus (GEO) under accession number GSE147529. The following figures have associated raw data: Figures 1a–c, 2b–h, 3d–i, 4a, 4b, 4d–f, 4h, 4i, and 5b–f; Supplementary Figures 1a–g, 2b–h, 3f, 3g, 3i, 4b–d, 4f and 5b–i. For gel Source data, see Supplementary Data 1.

Field-specific reporting

Please select the one below that is the best fit for your research. If you are not sure, read the appropriate sections before making your selection.

[x] Life sciences   [ ] Behavioural & social sciences   [ ] Ecological, evolutionary & environmental sciences

For a reference copy of the document with all sections, see nature.com/documents/nr-reporting-summary-flat.pdf

Life sciences study design

All studies must disclose on these points even when the disclosure is negative.

Sample size	Statistical methods were used to predetermined sample size in vitro and in vivo analyses. We designed in vivo experiments to ensure that minimum number of mice are used to obtain biologically significant results.
Data exclusions	No data was excluded from analyses.
Replication	All findings were replicated in a at least four independent experiments performed under identical conditions.
Randomization	For in vitro experiments, cultures were randomly chosen for different treatments and experiments were performed multiple times.<br>For mouse experiments, mice were randomly assigned by a technician blinded to the experimental design. Male mice were selected for the experiment in order to avoid concerns about hormonal effect in female mice. After the selection, mice for the DMM surgery, or intra-articular injection were allocated randomly without subjective judgement.
Blinding	Investigators were blinded to the cartilage destruction analysis, SBP thickness analysis, ratio of hyaline and calcified cartilage analysis of mice during histological analysis and scoring.

Reporting for specific materials, systems and methods

We require information from authors about some types of materials, experimental systems and methods used in many studies. Here, indicate whether each material, system or method listed is relevant to your study. If you are not sure if a list item applies to your research, read the appropriate section before selecting a response.

Materials & experimental systems

n/a	Involved in the study	
[ ]	[x]	Antibodies
[ ]	[x]	Eukaryotic cell lines
[x]	[ ]	Palaeontology
[ ]	[x]	Animals and other organisms
[ ]	[x]	Human research participants
[x]	[ ]	Clinical data

Methods

n/a	Involved in the study	
[x]	[ ]	ChIP-seq
[x]	[ ]	Flow cytometry
[x]	[ ]	MRI-based neuroimaging

Antibodies

Antibodies used	Immunohistochemistry<br>OSCAR (1:200; Santa Cruz Biotechnology; sc34235; Lot#E2115)<br>OSCAR (1:200; Biorbyt; orb185679; Lot#BR5396)<br>TRAIL (1:200; R&D system; AF1121; Lot#GNX021704A)<br>OPG (1:200; Santa Cruz Biotechnology; sc8468; Lot#D201)<br>MMP3 (1:50; Abcam; ab53015; Lot#GR3228789-8)

	MMP13 (1:25; Abcam; ab51072; Lot#GR3244070-7)<br>COL2A1 (1:50; Sigma-Aldrich; MAB8887; Lot#3215502<br>Aggrecan (1:100; Abcam; ab1031; Lot#3253326)<br>ADAMTS5 (1:50; GeneTex; gtx100332; Lot#39568)<br><br>Western Blotting<br>OSCAR (1:1000; Thermo Fisher Scientific; PA5-47171; Lot#TK2675474)<br>TRAIL (1:1000; Abcam; ab10516; Lot#3206951,#3268382-3)<br>OPG (1:1000; Santa Cruz Biotechnology; sc8468; Lot#D201)<br>OPG (1:1000; Abcam; ab183910; Lot#3199207-19)<br>GAPDH (1:1000; Santa Cruz Biotechnology; sc32233; Lot#K3016)<br>B-Actin (1:1000; Santa Cruz Biotechnology; sc47778; Lot#C1919)
Validation	All antibodies used in this study were validated by the suppliers as follows;<br>OSCAR (1:200; Santa Cruz Biotechnology; sc34235; Lot#E2115) for IHC; species (Mouse, Human, Rat), application (WB, IF, ELISA)<br>OSCAR (1:200; Biorbyt; orb185679; Lot#BR5396) for IHC; species (Mouse, Human, Rat), application (ELISA,ICC,IF,IHC-P)<br>manufacturer's website (https://www.biocompare.com/9776-Antibodies/5801021-OSCAR-antibody/)<br>OSCAR (1:1000; Thermo Fisher Scientific; PA5-47171; Lot#TK2675474) for WB; species (Mouse), application (WB)<br>manufacturer's website (https://www.thermofisher.com/antibody/product/OSCAR-Antibody-Polyclonal/PA5-47171)<br>TRAIL (1:200; R&D system; AF1121; Lot#GNX021704A) for IHC; species (Mouse), application (IHC-P, ELISA, WB)<br>manufacturer's website (https://www.rndsystems.com/products/mouse-trail-tnfsf10-antibody_af1121)<br>TRAIL (1:200; Abcam; ab10516; Lot#3206951-1) for WB; species (Mouse, Human), application (IHC-P, Neutralising, Inhibition Assay, ELISA, WB)<br>manufacturer's website (https://www.abcam.com/trail-antibody-7541111-ab10516)<br>OPG (1:200; Santa Cruz Biotechnology; sc8468; Lot#D201) for IHC, WB; species (Mouse, Rat, Human), application (WB, IP, IF, IHC-P)<br>manufacturer's website (https://www.scbt.com/scbt/ko/product/opg-antibody-n-20)<br>OPG (1:1000; Abcam; ab183910; Lot#3199207-19) for WB; species (Mouse, Human), application (WB, IHC-P)<br>manufacturer's website (https://www.abcam.com/osteoprotegerin-antibody-ab183910.html)<br>MMP3 (1:50; Abcam; ab53015; Lot#GR3228789-8) for IHC; species (Mouse, Rat, Human), application (IHC-P, WB, ELISA, ICC/IF)<br>manufacturer's website (https://www.abcam.com/mmp3-antibody-ab53015.html)<br>MMP13 (1:25; Abcam; ab51072; Lot#GR3244070-7) for IHC; species (Human), application (WB, Flow Cyt, ICC/IF, IHC-Fr, IHC-P or IP)<br>manufacturer's website (https://www.abcam.com/mmp13-antibody-ep1263y-ab51072.html)<br>COL2A1 (1:50; Sigma-Aldrich; MAB8887; Lot#3215502) for IHC; species (Chicken, Human, Mouse, Salamander), application (IF, IHC, WB)<br>manufacturer's website (https://www.merckmillipore.com/KR/ko/product/Anti-Collagen-Type-II-Antibody-clone-6B3,MM_NF-MAB8887)<br>Aggrecan (1:100; Sigma-Aldrich; ab1031;Lot#3253326) for IHC; species (Mouse), application (IF, IHC-P, IP, WB)<br>manufacturer's website (https://www.merckmillipore.com/KR/ko/product/Anti-Aggrecan-Antibody,MM_NF-AB1031)<br>ADAMTS5 (1:50; GeneTex; gtx100332; Lot#39568) for IHC; species (Human, Mouse), application (WB, IHC-P)<br>manufacturer's website (https://www.genetex.com/Product/Detail/ADAMTS5-antibody/GTX100332)<br>GAPDH (1:1000; Santa Cruz Biotechnology; sc32233; Lot#K3016) for WB; species (Mouse, Rat, Human, Rabbit), application (WB, IHC, IF)<br>manufacturer's website (https://www.scbt.com/scbt/product/gapdh-antibody-6c5)<br>B-Actin (1:1000; Santa Cruz Biotechnology; sc47778; Lot#C1919) for WB; species (mouse, rat, human, avian, bovine, canine, porcine, rabbit, Dictyostelium discoideum, Physarum polycephalum), application (WB, IP, IF, IHC, ELISA)<br>manufacturer's website (https://www.scbt.com/ko/p/beta-actin-antibody-c4)

Eukaryotic cell lines

Policy information about cell lines

Cell line source(s)	HEK293F from Thermo Fisher Scientific (Cat No. R79007)
Authentication	None of these cell lines were authenticated by us.
Mycoplasma contamination	We confirmed that cell lines used were negative for mycoplasma contamination.
Commonly misidentified lines<br>(See ICLAC register)	No commonly misidentified cell lines were used.

Animals and other organisms

Policy information about studies involving animals; ARRIVE guidelines recommended for reporting animal research

Laboratory animals	The study involved the following laboratory animals:<br>C57BL/6J male mice (purchased from Jackson laboratory and bred in the unit)<br>OSCAR knock out mice (embryos were a gift from Prof Yongwon Choi and mice were bred in house).<br>All mice used in experimental studies were males aged 8-12 weeks old weighing between 20-25g.
Wild animals	This study did not involve wild animals.
Field-collected samples	This study did not involve animals collected from the field.
Ethics oversight	All experiments were approved by the institutional committee and conformed to the guidelines and laws set by the Laboratory Animal Genomic Center, Ewha, South Korea.

Note that full information on the approval of the study protocol must also be provided in the manuscript.

Human research participants

Policy information about studies involving human research participants

Population characteristics	International Cartilage Repair Society (ICRS) grade 4 cartilage tissues were obtained from patients with osteoarthritis aged from 63 to 74 years during total knee replacement surgery. The cartilage tissue offered by these patients were grouped by intact and damaged region for the following analysis. No patients with osteoarthritis had rheumatoid arthritis, metabolic diseases, or other inflammatory diseases at the time of surgery.
Recruitment	Patients undergoing surgery were recruited for offering cartilage on an 'as available' basis in Gangnam Severance Hospital. The protocol, including the use of cartilage, was approved by the Ethics Committee of Yonsei University Gangnam Severance Hospital, Seoul, Korea. All participating patients provided signed informed consent.
Ethics oversight	Human subjects research was performed according to the Institutional Review Boards at Yonsei University Gangnam Severance Hospital.

Note that full information on the approval of the study protocol must also be provided in the manuscript.
